# Biomechanical Assessment of Endodontically Treated Molars Restored by Endocrowns Made from Different CAD/CAM Materials

**DOI:** 10.3390/ma16020764

**Published:** 2023-01-12

**Authors:** Mhd Ayham Darwich, Abeer Aljareh, Nabil Alhouri, Szabolcs Szávai, Hasan Mhd Nazha, Fabian Duvigneau, Daniel Juhre

**Affiliations:** 1 Faculty of Biomedical Engineering, Al-Andalus University for Medical Sciences, Tartous, Syria; 2 Faculty of Technical Engineering, University of Tartous, Tartous, Syria; 3 Faculty of Dentistry, Damascus University, Damascus, Syria; 4 Faculty of Mechanical Engineering and Informatics, University of Miskolc, 3515 Miskolc, Hungary; 5 Faculty of Mechanical Engineering, Institute of Mechanics, Otto Von Guericke University Magdeburg, Universitätsplatz 2, 39106 Magdeburg, Germany

**Keywords:** dental crowns, finite element analysis, CAD-CAM, lithium disilicate, ceramics, zirconium oxide

## Abstract

The aim of this study was to evaluate the deflection and stress distribution in endodontically treated molars restored by endocrowns from different materials available for the computer-aided design/computer-aided manufacturing (CAD/CAM) technique using three-dimensional finite element analysis. The models represented extensively damaged molars restored by endocrowns from the following materials: translucent zirconia; zirconia-reinforced glass ceramic; lithium disilicate glass ceramic; polymer-infiltrated ceramic network (PICN) and resin nanoceramic. Axial and oblique loadings were applied and the resulting stress distribution and deflection were analyzed. The Mohr–Coulomb (MC) ratio was also calculated in all models. The translucent zirconia endocrown showed the highest stress concentration within it and the least stress in dental structures. The resin nanoceramic model was associated with the greatest stress concentration in dental tissues, followed by the PICN model. Stress was also concentrated in the distal region of the cement layer. The MC ratio in the cement was higher than 1 in the resin nanoceramic model. Oblique loading caused higher stresses in all components and greater displacement than axial loading, whatever the material of the endocrown was. The translucent zirconia model recorded deflections of enamel and dentin (38.4 µm and 35.7 µm, respectively), while resin nanoceramic showed the highest stress concentration and displacement in the tooth–endocrown complex.

## 1. Introduction

The restoration of endodontically treated teeth is still a real challenge, particularly teeth with extensively damaged coronal tissues [1]. Although various types of intraarticular posts have been used widely to provide retention for the core material [1,2], they might weaken the restored teeth due to the additional removal of sound dental structures [3]. Moreover, the preparation of the post space may increase the risk of root perforation and root fracture [1].

Taking advantage of the increasing interest in minimally invasive treatment as well as the evolution of dental materials manufacturing, endocrowns had been introduced as a more conservative approach to rehabilitate endodontically treated teeth with large loss of coronal tissues [4,5]. It was first introduced by Pissis in 1995 as a monoblock ceramic restoration. This restoration is composed of a monoblock ceramic crown anchored on the cavity margins with an extension to the pulp chamber. Mechanical and micromechanical retention can be provided from the pulp chamber walls and the adhesive cement, respectively [6]. Endocrowns preserve dental tissues and reduce the risk of tooth fracture when compared to conventional treatment by post and core systems [7]. They also save costs and time, as they minimize the required clinical and technical procedures [8]. Furthermore, endocrowns are associated with less stress concentration and higher fracture strength than conventional crowns supported by different types of posts and cores [6,9,10].

The computer-aided design/computer-aided manufacturing (CAD/CAM) technique has been used widely in dentistry [11]. This manufacturing technology reduces needed time and procedures. Furthermore, its restorations are more accurate, with better marginal adaptation than restorations fabricated by conventional techniques [12]. Numerous CAD/CAM materials are utilized to fabricate various restorations, including endocrowns [5,11,12]. 3Y-TZP zirconia (yttrium-cation-doped tetragonal zirconia polycrystals: 2–3% mol Y2O3) [13] is known for its highest strength and toughness among dental ceramics [14]. However, the opacity of traditional zirconia has led to the generation of more translucent zirconia by reducing the alumina amount to 0.05% by weight and increasing the yttria content to 4% mol (4Y-PSZ: yttria partially stabilized zirconia), 5% mol (5Y-PSZ) and recently to 8% mol (e.g., DD Bio ZX2, Dental Direkt, Spenge, Germany) [13,14,15]. This new generation of zirconia has allowed for the production of full anatomical zirconia restorations with appropriate esthetic appearances, using CAD/CAM manufacturing technology [13]. On the other hand, lithium disilicate glass ceramic (LDS) is another example of ceramics that shows high mechanical and esthetic properties and could be fabricated by the CAD/CAM technique (e.g., IPS e.max CAD, Ivoclar Vivadent, Ellwangen, Germany) [16]. Thanks to the needle-shaped crystals that form within the glass ceramic during crystallization, the flexural strength and fracture toughness of the material are doubled [17].

Various types of CAD/CAM materials have been produced due to the combination of different components such as glass ceramic, zirconia crystals, and resin matrix. Polymer-infiltrated ceramic network (PICN) material has been introduced as a result of infiltrating a ceramic scaffold (86% by weight) with a resin network (14% by weight) (e.g., VITA ENAMIC, VITA Zahnfabrik, Bad Säckingen, Germany) [18]. This composition offers many benefits. For instance, PICN, which is described as a hybrid ceramic, is much easier to mill by the CAD/CAM technique compared to other ceramics. Moreover, its tendency to brittle fracture is lower than pure ceramics [18]. Zirconia-reinforced lithium silicate glass ceramic (ZLS) (e.g., VITA SUPRINITY, Vita Zahnfabrik, Germany) is another CAD/CAM material that is fabricated by adding zirconia crystals (10% by weight) to glass ceramic [19]. Not only does ZLS have high mechanical and esthetic properties due to its structure, but it also provides better milling and polishing procedures than LDS, according to the manufacturer [19]. Resin nanoceramic (RNC) (e.g., Lava Ultimate, 3M ESPE, St. Paul, MN, USA) was suggested to be classified as a type of resin-matrix ceramic as it contains 80% by weight silica and zirconia nanoparticles with a highly cured resin matrix [20,21]. This formulation allows for the fabrication of restorations with high strength and high polish retention. It also allows for faster manufacturing procedures [20]. 

The wide variation of available materials in the market, which are all alleged to be characterized by high esthetic and physical properties according to the manufacturers [16,18,19,20], makes it difficult to choose a suitable restorative material depending on its biomechanical behavior. Furthermore, there is no crucial conclusion about endocrown materials. An earlier study found that the facture load and the flexural strength of lithium disilicate ceramic (mean value 0.4 KN and 271.6 MPa, respectively) were the highest among studied materials (resin nanoceramic, feldspathic ceramic and PICN). It also found that the strength of feldspathic ceramic (137.8 MPa) was less than the strength of resin nanoceramic (164.3 MPa) [11]. However, another study concluded that resin nanoceramic endocrowns had significantly higher fracture resistance (1583.28 N) than LDS and feldspathic ceramic endocrowns (1340.92 N and 1368.76 N, respectively) [8]. Moreover, Aktas et al. [22] showed no differences in the mechanical failure of endocrowns from alumina-silicate, zirconia-reinforced lithium silicate ceramic and PICN. In addition to the conflicting results about endocrown materials, there is a lack of studies about the behavior of endocrowns from translucent zirconia. Sahebi et al. [23] concluded that translucent zirconia endocrowns showed higher fracture strength with lower retention than zirconium lithium silicate endocrowns. While a previous study found that resin composite endocrowns were a reliable approach to restoring endodontically treated teeth compared to lithium disilicate and translucent zirconia endocrowns or crowns with conventional posts [24], another study found that higher fracture strength was seen in lithium disilicate ceramic endocrowns, whereas translucent zirconia endocrowns showed more catastrophic failure types [25]. 

The biomechanical behavior of restorative materials could play an important role in choosing the suitable material for endocrowns to rehabilitate molars with excessively damaged coronal structures. The conflicting conclusions of previous studies about endocrown materials led us to carry out this study. To our knowledge, the present investigation is one of the first studies to assess stress distribution in molars restored by endocrowns from translucent zirconia. Thus, this study aimed to evaluate the biomechanical behavior of endocrowns from translucent zirconia and compare it with endocrowns from other CAD/CAM materials, using finite element analysis (FEA).

## 2. Materials and Methods

### 2.1. FEA Modeling

**Figure 1:**
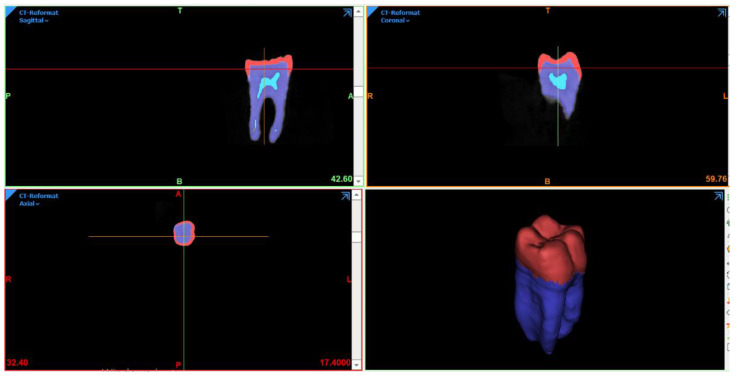
The isolated dental components.

At first, mandibular molar was scanned using cone-beam computed tomography imaging technique (CBCT); (Pax-i3D Green; Vatech, Gyeonggi-do, Republic of Korea). Then, image slices were imported to Mimics 21.0 software (Materialise NV Technologielaan, Leuven, Belgium), and 186 slices were selected from a total of 416 slices. Separate masks were created to isolate dental tissues—enamel; dentin and pulp—in each slice of the image (Figure 1), depending on Hounsfield Units (HU) in the CBCT image (6830–7000 HU for enamel, 4179–6198 HU for dentin and 3499–4617 HU for pulp). 

**Figure 2:**
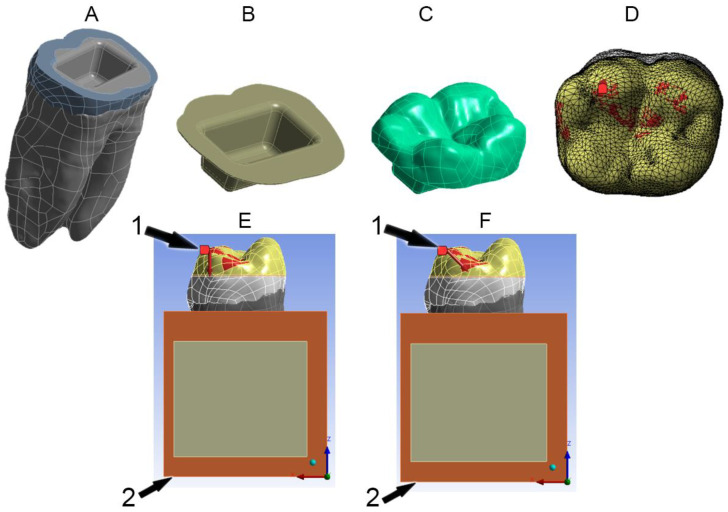
(**A**): dental tissues; (**B**): cement layer; (**C**): endocrown; (**D**): loading points on endocrown; (**E**): axial loading; (**F**): oblique loading. 1: applied force and 2: fixed support (inferior surface of cortical bone).

Utilizing 3Matic software (Materialise NV Technologielaan, Leuven, Belgium), the periodontal ligament was created by the offset feature with a thickness of 0.25 mm. The 3D objects were exported as STL files to Geomagic Studio (Geomagic Inc., Morrisville, NC, USA) to refine them and generate NURBS (non-uniform rational B-spline) models in IGES format. Powershape Ultimate software (Autodesk Inc., San Rafael, CA, USA) was used then to convert the components to solids and export them in Parasolid (x.t) format. Afterwards, SolidWorks software (Dassault Systèmes SolidWorks Corporation, Waltham, MA, USA) was used to create the preparation design and the surrounding bones. Cortical and trabecular bones were created according to the dimensions mentioned in the literature [26,27], as shown in Figure 2A.

### 2.2. Preparation Design

Using SolidWorks, coronal tissues were cut 2 mm above the cementoenamel junction (CEJ) to simulate the extensive loss of coronal structures [5,28,29]. The occlusal margins were flat to represent butt joint preparation (Figure 2A) [5,29]. The pulp chamber was 3 mm in depth [30] with rounded angles and 10° internal wall divergence [4,28,29], (Figure 2B). A 0.1 mm thickness of the cement layer was also represented [10,31], as shown in Figure 2B. 

Boolean operations were performed using Powershape Ultimate software to obtain the restorations and prepared dental structures separately. Five different CAD/CAM materials were used to represent the endocrown. Therefore, the models were: translucent zirconia endocrown (E-Z); zirconia-reinforced lithium silicate ceramic endocrown (E-S); lithium disilicate glass ceramic endocrown (E-E); polymer-infiltrated ceramic network endocrown (E-P) and resin nanoceramic endocrown (E-L). Table 1 shows the models and their materials.

Meshing was performed for each model by generating a mesh of tetrahedral quadratic elements. Based on mesh sensitivity analysis, the optimized number of elements is reported in Table 2, based on 1% convergence tolerance in model output for each component.

### 2.3. Material Properties

All materials and components were assumed to be linearly elastic, isotropic and homogenous. All components were also assumed to be bonded to each other in the model. Table 3 summarizes the mechanical properties of materials and structures taken from the literature [32,33,34,35,36,37,38].

### 2.4. Boundary Conditions

The inferior surface of the cortical bone was fixed in all directions. Axial and oblique loadings of 600 N were separately applied on the occlusal contact points—the buccal cusps tips, the central fossa and the distal marginal ridge [35]—as shown in Figure 2E. The axial loading was applied parallelly to the longitudinal axis of the tooth as a normal force on the molars, whereas the other loading was applied 45 degrees to the longitudinal axis of the tooth. The oblique loading simulated the force on molars during the closing phase of the mastication cycle [35]. Used in many studies to evaluate stresses in diverse restorative materials [39,40], the equivalent von Mises stresses are evaluated in molars restored by endocrowns from different CAD/CAM materials. Von Mises stress theory, based on the distortion energy theory in engineering, is a combination of the three principal stresses (σ1, σ2 and σ3) in the studied field. If any of these stresses reach a critical value related to the property of the material, the material begins to fail [41]. Total deformation was analyzed to determine the total displacement in the endocrown as well as the dental structures. Maximum principal (tensile) stress, which is used as an index of failure in brittle materials, and minimum principal (compressive) stress were also calculated in the restorations and the tooth [31,35,39,40,42]. Depending on the peak values of maximum and minimum principal stresses [43], the Mohr–Coulomb ratio was calculated in each model. Maximum principal stress theory is used to predict failure in brittle materials; when this stress exceeds the ultimate strength, failure would occur in the material [44]. When the maximum principal stress exceeds the ultimate tensile strength of the material, or when the minimum principal stress exceeds the compressive strength of the material, the material is predicted to fail [43]. Failure is also predicted when the combination of the maximum principal stress and the minimum principal stress equals or exceeds the ultimate strengths [43]. The Mohr–Coulomb theory can predict failure in brittle materials, and its formula is given as follows:

(1) $$\sigma_{\mathrm{MC}}=\frac{\sigma_{\mathrm{MAX}}}{\mathrm{UTS}}+\frac{\sigma_{\mathrm{MIN}}}{\mathrm{UCS}}$$

where UTS is the ultimate tensile strength and UCS is the ultimate compressive strength of the material, while σ_MAX_ and σ_MIN_ are the maximum and minimum principal stresses, respectively [43].

## 3. Results

The results obtained from the FEA are shown in Figure 3, Figure 4, Figure 5, Figure 6, Figure 7, Figure 8, Figure 9, Figure 10, Figure 11 and Figure 12. The color scale in color maps ranges from purple and red (the highest stresses or deflection) to blue (the lowest stresses or deflection) in each model.

### 3.1. Endocrowns

**Figure 3:**
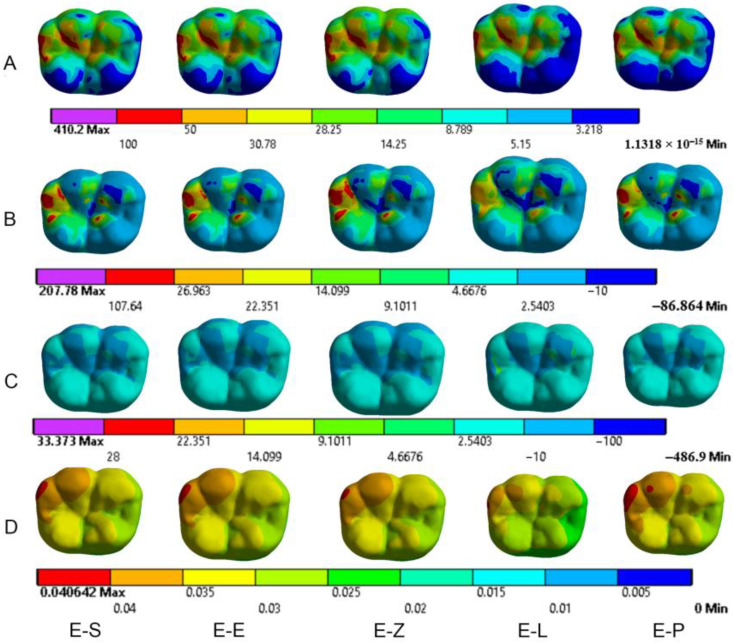
Stress distribution and displacement of endocrowns in all models under axial loading. (**A**) von Mises stress, (**B**) maximum principal stress, (**C**) minimum principal stress and (**D**) displacement. E-S: zirconia-reinforced lithium silicate ceramic model, E-E: lithium disilicate glass ceramic model, E-Z: translucent zirconia model, E-L: resin nanoceramic model and E-P: polymer-infiltrated ceramic network model.

**Figure 4:**
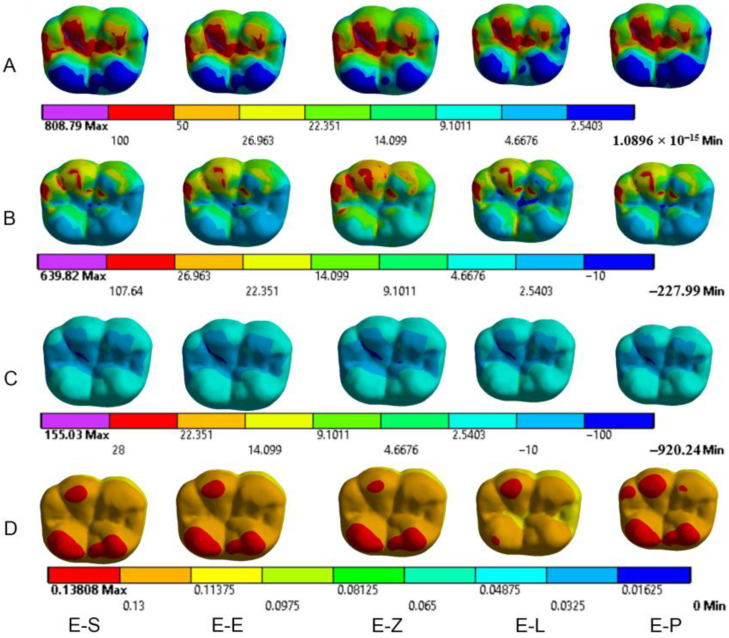
Stress distribution and displacement of endocrowns in all models under oblique loading. (**A**) von Mises stress, (**B**) maximum principal stress, (**C**) minimum principal stress and (**D**) displacement. E-S: zirconia-reinforced lithium silicate ceramic model, E-E: lithium disilicate glass ceramic model, E-Z: translucent zirconia model, E-L: resin nanoceramic model and E-P: polymer-infiltrated ceramic network model.

**Figure 5:**
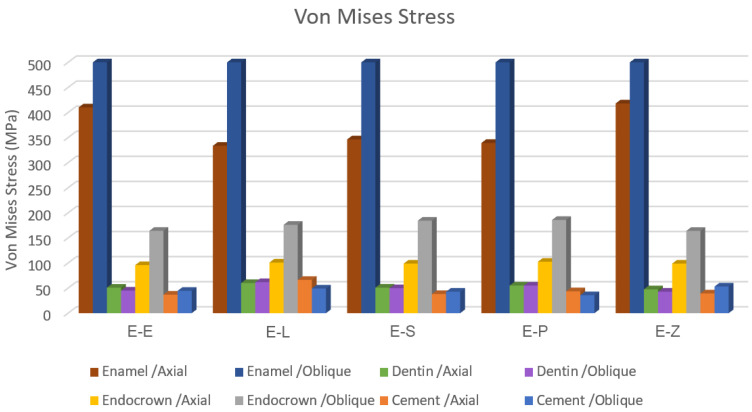
The maximum values of von Mises stress (MPa). E-Z: translucent zirconia model, E-P: polymer-infiltrated ceramic network model, E-S: zirconia-reinforced lithium silicate ceramic model, E-L: resin nanoceramic model and E-E: lithium disilicate glass ceramic model.

Color maps of stress distribution in endocrowns are shown in Figure 3 and Figure 4. The von Mises stresses in endocrowns when axial and oblique loadings were applied are cited in Figure 5. Von Mises stresses are mostly concentrated in the distal marginal ridge of endocrowns in all models. Furthermore, oblique loading causes much higher stress concentrations in endocrowns than axial loading (Figure 3 and Figure 4). Under both loadings, stress concentration in endocrowns is the highest in the E-Z model, followed by the E-S and E-E models, whereas the lowest stresses in endocrowns are in the E-L model.

**Figure 6:**
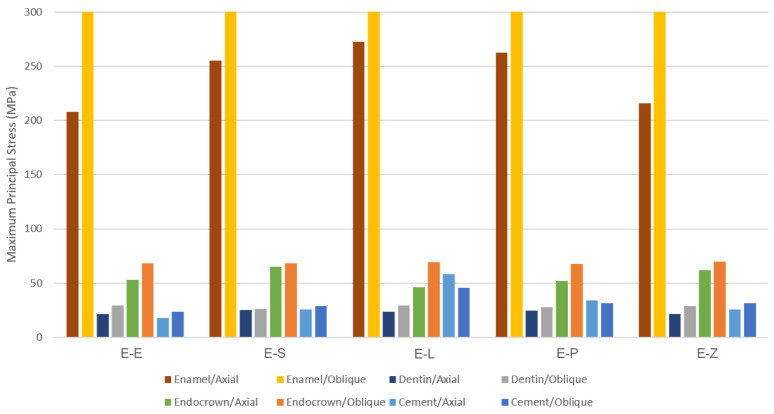
The maximum values of maximum principal stresses (MPa). E-E: lithium disilicate glass ceramic model, E-S: zirconia-reinforced lithium silicate ceramic model, E-L: resin nanoceramic model, E-P: polymer-infiltrated ceramic network model and E-Z: translucent zirconia model.

Maximum principal (tensile) stress is concentrated in the distal marginal ridge of endocrowns under axial loading (Figure 3 and Figure 4). The E-L endocrown shows the least stress concentration and the lowest value of tensile stress (46.3 MPa). The greatest tensile stress concentration is in the E-Z endocrown under both loadings. The E-Z endocrown also shows the highest tensile stress (69.7 MPa) when oblique loading is applied. However, the values of tensile stress in endocrowns from all materials are similar under oblique loading (Figure 6).

**Figure 7:**
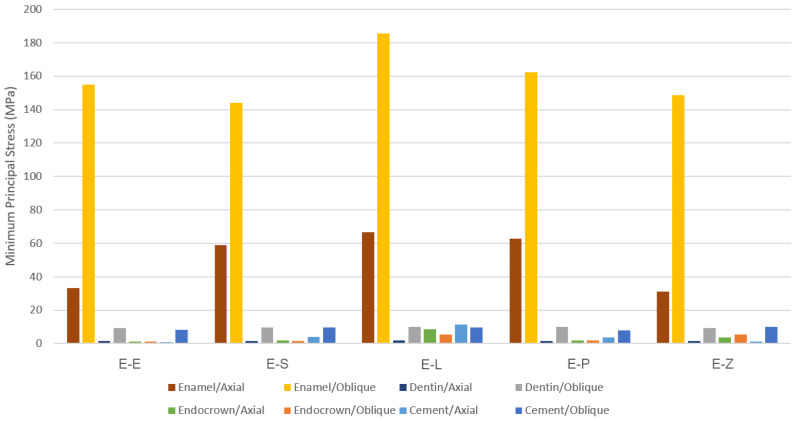
The maximum values of minimum principal stresses (MPa). E-E: lithium disilicate glass ceramic model, E-S: zirconia-reinforced lithium silicate ceramic model, E-L: resin nanoceramic model, E-P: polymer-infiltrated ceramic network model and E-Z: translucent zirconia model.

The minimum principal (compressive) stress concentration is similar in all endocrowns regardless of the loading direction. The highest value of compressive stress is seen in the E-L and E-Z endocrowns under axial loading, while the lowest values are seen in the E-E endocrown under axial and oblique loadings (1.4 MPa and 1.3 MPa), respectively, as shown in Figure 7.

**Figure 8:**
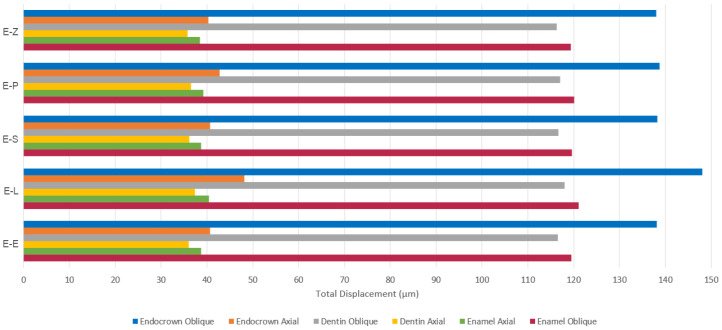
Maximum values of deflection (µm) of endocrowns, dental tissues and cement layer. E-Z: translucent zirconia model, E-P: polymer-infiltrated ceramic network model, E-S: zirconia-reinforced lithium silicate ceramic model, E-L: resin nanoceramic model and E-E: lithium disilicate glass ceramic model.

The values of the Mohr–Coulomb (MC) ratio in endocrowns from all materials under axial and oblique loadings are shown in Table 4. The values under oblique loading are higher than axial loading. All values are lower than 1 in all models. However, the E-Z endocrown shows the lowest MC ratio, while the EL and E-P endocrowns show the highest MC ratios. The total displacement of endocrowns for most of the models is in the same range, except for the E-L endocrown, which shows the highest displacement (48.1 µm under axial loading and 148.0 µm under oblique loading). The E-Z endocrown records the lowest values of displacement (40.2 µm under axial loading and 138.0 µm under oblique loading), as shown in Figure 8. The maximum displacement occurs in the occlusal surface of the endocrown whatever the material and the direction of the loading were (Figure 3 and Figure 4).

**Figure 9:**
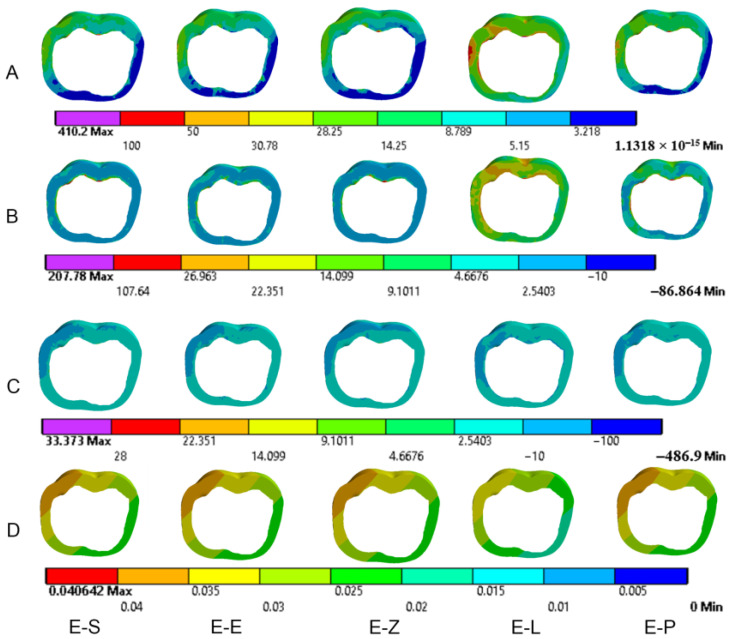
Stress distribution and displacement of enamel in all models under axial loading. (**A**) von Mises stress, (**B**) maximum principal stress, (**C**) minimum principal stress and (**D**) displacement. E-S: zirconia-reinforced lithium silicate ceramic model, E-E: lithium disilicate glass ceramic model, E-Z: translucent zirconia model, E-L: resin nanoceramic model and E-P: polymer-infiltrated ceramic network model.

**Figure 10:**
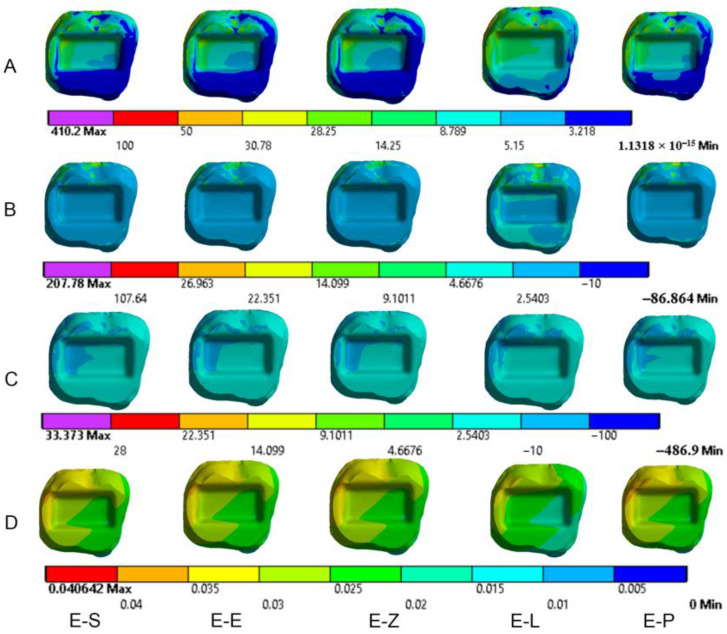
Stress distribution and displacement of dentin in all models under axial loading. (**A**) von Mises stress, (**B**) maximum principal stress, (**C**) minimum principal stress and (**D**) displacement. E-S: zirconia-reinforced lithium silicate ceramic model, E-E: lithium disilicate glass ceramic model, E-Z: translucent zirconia model, E-L: resin nanoceramic model and E-P: polymer-infiltrated ceramic network model.

**Figure 11:**
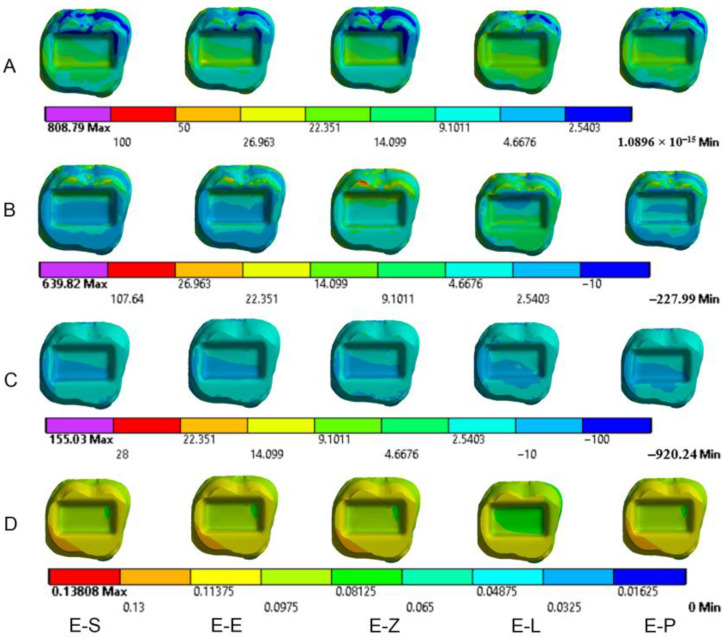
Stress distribution and displacement of dentin in all models under oblique loading. (**A**) von Mises stress, (**B**) maximum principal stress, (**C**) minimum principal stress and (**D**) displacement. E-S: zirconia-reinforced lithium silicate ceramic model, E-E: lithium disilicate glass ceramic model, E-Z: translucent zirconia model, E-L: resin nanoceramic model and E-P: polymer-infiltrated ceramic network model.

Von Mises stress is concentrated in the distal surface of enamel in all models when axial loading is applied (Figure 9). Oblique loading is associated with greater stress concentration in the buccal part of the occlusal surface of enamel compared to axial loading. It also causes more stress concentration in the lingual wall of the pulp chamber (Figure 10 and Figure 11). Moreover, oblique loading causes higher stress values in enamel and dentin than in axial loading (Figure 5). Not only does the E-L model record the highest values of von Mises stresses in dentin (60.1–61.8 MPa) under axial and oblique loadings (Figure 5), but it also shows the greatest stress concentration in dental tissues, followed by the E-P model. The lowest stress concentration in enamel and dentin is found in the E-Z model (Figure 9, Figure 10 and Figure 11).

### 3.2. Dental Structures

Maximum principal (tensile) stress is concentrated in the distal region of enamel under axial loading (Figure 9). Oblique loading causes higher tensile stresses in dental structures than axial loading in all models (Figure 6 and Figure 11). In addition, greater stress is concentrated in the occlusal surface under oblique loading. The highest maximum principal stress concentration in dental tissues is in the E-L model whatever the direction of the loading was. 

Oblique loading increases the concentration of minimum principal stress in the lingual part of the occlusal surface in all models. The highest values of compressive stress in dental tissues are in the E-L model under both loadings (Figure 7).

The maximum deflection of dental tissues is in the distal buccal area of the preparation in all models under axial loading (Figure 9). It is greater in the lingual part of the coronal tissues when oblique loading was applied (Figure 11). The E-L model shows the highest displacement of dental structures under both loadings, followed by the E-P model, the E-S model, the E-E model and finally the E-Z model. The last model recorded the lowest deflection of enamel and dentin (38.4 µm and 35.7 µm, respectively, under axial loading), as shown in Figure 8.

### 3.3. Cement

**Figure 12:**
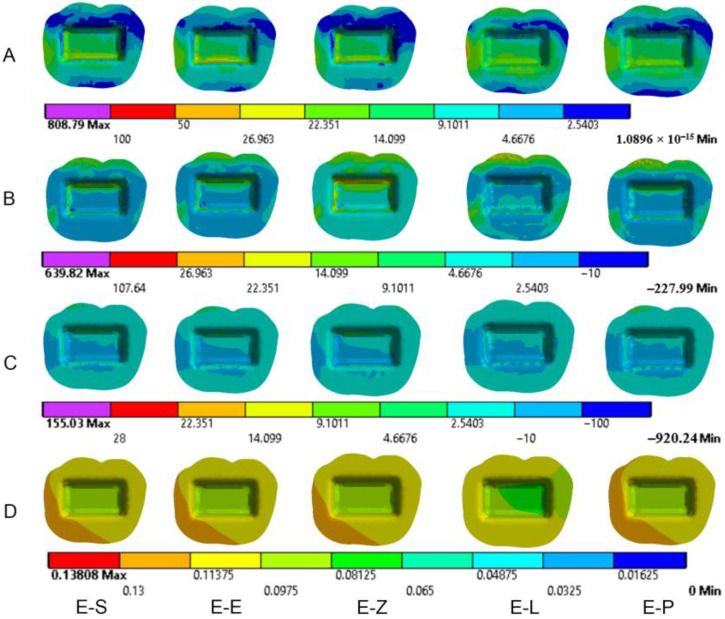
Stress distribution and displacement of cement in all models under oblique loading. (**A**) von Mises stress, (**B**) maximum principal stress, (**C**) minimum principal stress and (**D**) displacement. E-S: zirconia-reinforced lithium silicate ceramic model, E-E: lithium disilicate glass ceramic model, E-Z: translucent zirconia model, E-L: resin nanoceramic model and E-P: polymer-infiltrated ceramic network model.

The values and the distribution of von Mises stress in the cement layer are shown in Figure 5 and Figure 12A. Stress is concentrated in the distal region of the cement layer. Oblique loading is associated with higher stresses in the cement than axial loading (Figure 6 and Figure 12). The E-Z model shows the lowest stress concentration in the cement layer. In contrast, the greatest stress concentration in the cement layer is seen in the E-L model, followed by the E-P model.

The highest values of tensile stress in the cement layer are in the E-L model (58.0 MPa, 45.5 MPa), whereas the lowest values are in the E-E model (18.0 MPa, 23.6 MPa) under axial and oblique loadings, respectively (Figure 6). Oblique loading causes higher stress concentration, particularly in the buccal area of the cement in E-L and E-P models (Figure 12). 

More minimum principal stress is concentrated in the buccal area of the cement under oblique loading (Figure 12). Although the values of compressive stresses are similar under oblique loading in all models, the highest values under axial loading are seen in the E-E model (1.0 MPa) as shown in Figure 7. The Mohr–Coulomb ratio in cement is higher than 1 in the E-L (resin nanoceramic) model (Table 5). The values of the MC ratio are lower than 1 in the cement layer in the other models.

The values of deflection range from 38.647 µm to 41.019 µm under axial loading. However, these values increase to approximately 120.6 µm when oblique loading is applied (Figure 8). The displacement is the highest in the lingual area of the cement under oblique loading (Figure 12).

## 4. Discussion

Dentists still face difficulty when restoring endodontically treated posterior teeth with extensively damaged coronal tissues [45]. Endocrowns offer a good choice to rehabilitate such teeth [45], as they minimize contact interfaces between different materials within the restoration system and reduce the need for additional macroretentive features in comparison with the conventional post and core approach [29]. It could also be the best restoration when the interocclusal space is limited or the clinical crown length is inadequate [8]. Taking advantage of the development of CAD/CAM technology and the expansion of its materials, endocrowns from various CAD/CAM materials have been manufactured recently with more accurate marginal adaptation and high fracture strength [5,12].

Finite element analysis (FEA) is an effective tool that has been used widely to evaluate stress distribution in complex systems, such as tooth–restoration, and to expect their behavior under various conditions [40]. Using the Mohr–Coulomb ratio, which is based on maximum and minimum principal stresses, failure is expected if this ratio exceeds 1 [43]. The von Mises stress was evaluated in all models in this study. Minimum and maximum principal stresses were utilized to calculate stresses in endocrowns and restored teeth, too. The Mohr–Coulomb ratio and total displacement were also assessed in each model to evaluate the biomechanical behavior of endocrowns from different CAD/CAM materials that restore mandibular molars. Posterior teeth are subjected to various functional and parafunctional forces in different directions [46]. Normal bite forces on molars range from 520 N to 800 N [39]. Thus, 600 N was applied in two directions on the occlusal surface of the molar model in this FEA study.

Not surprisingly, oblique loading caused higher stress concentration and greater values of von Mises, tensile and compressive stresses in all structures than axial loading. It also increased the values of total displacement by approximately 32% in all components of the models. This finding, which is in line with other studies [31,39] confirms the harmful effects of nonaxial forces on the tooth–restoration complex [47]. Nonaxial loadings, including oblique loading, resolve to their axial and horizontal components. The axial component distributes stresses along the longitudinal axis of the structure subjected to the loading, while the horizontal component concentrates stresses in this structure.

While minimum principal (compressive) stress showed a similar pattern of distribution in all endocrowns, the concentration of von Mises stress and maximum principal stress in endocrowns was seen in the distal marginal ridge. The maximum deflection of the endocrown was in the occlusal surface; in the distal buccal cusp under axial loading and in the lingual cusps under oblique loading, respectively. This result partially corresponds with the findings of Hasan et al. [48], who found that the maximum deflection occurred at the occlusal third of the endocrown. The displacement and stress concentration could indicate that cracks would initiate in the loading points, particularly the distal marginal ridge during clinical function [4,39]. Furthermore, this pattern of stress concentration might clarify why cracks and fractures started at these points in previous mechanical studies [4,31,39]. Even though Pérez-González et al. [49] concluded that von Mises theory is not an appropriate criterion for brittle materials, as they found that von Mises theory had predicted failure in the compressive areas rather than in tensile areas, von Mises theory in this study predicted failure in the same areas as tensile stress theory did in endocrowns, and in most of the tensile stress areas in the dental tissues.

The greatest values of minimum principal (compressive) stress were seen in endocrowns in the E-L model under both loadings. The concentration of compressive stress in resin nanoceramic endocrowns caused by the occlusal compressive loading could be explained by the elastic behavior of this material, which allows the material to withstand high compressive stresses before distortion. Nevertheless, the level of tensile stresses is a critical concern for the potential failure of the material [50]. Although the highest values of maximum principal stress were seen in endocrowns from translucent zirconia, maximum principal stress in endocrowns in all models did not exceed the ultimate tensile strength of studied materials whatever the direction of the loading was. The greatest von Mises and tensile stresses in endocrowns were found in translucent zirconia endocrowns (E-Z), while they were the lowest in the dental tissues in the E-Z model. In contrast, the E-L model (resin nanoceramic) showed the greatest stress concentration in dental structures and the lowest stresses in endocrowns. This result may be attributed to the elastic moduli of the studied materials. Material with high elastic modulus is more probable to concentrate stress within it rather than transmit stresses to the surrounding structures [4,39]. Furthermore, the E-Z model showed the smallest values of total displacement in all components and the lowest value of the Mohr–Coulomb ratio in the endocrown, while the E-L and E-P models were associated with the highest values of deflection and the highest values of Mohr–Coulomb ratio. This displacement could be due to the stiffness of the restorative material and its elastic modulus. Stiff materials, whose elastic moduli are high [32], cause less displacement of dental structures. Thus, endocrowns from such materials could provide greater protection for the residual dental tissues than endocrowns from materials whose elastic moduli are low [41,51]. Furthermore, the results showed that the highest displacement occurred in the lingual part of the occlusal surface in the dental structures under oblique loading. This could be due to the direction of the loading, which was applied at 45 degrees to the longitudinal axis from the buccal area towards the lingual area of the tooth. This displacement of the dental structures might result in tooth fracture [41]. The high deflection and stress concentration in the dental structures in the E-L model, which are restored by elastic material (resin nanoceramic), might explain why teeth restored by resin nanoceramic endocrowns showed lower fracture strength than teeth restored by LDS endocrowns in a previous study [47]. This finding could also clarify the reason for cracks that occurred in the residual dental tissues restored by endocrowns in a previous study [39].

Regarding stresses in the cement layer, tensile stress concentrated in the buccal area of the cement near margins in the E-L and E-P models. The highest values of von Mises and tensile stresses in the cement were also in the E-L model. This could be attributed to the low elastic modulus of resin nanoceramic, which leads to the concentration of more stresses in the neighboring structures, including the cement. The values of the Mohr–Coulomb ratio in cement in the E-L model were greater than 1 under both loadings. Moreover, the maximum principal stresses in the cement in the E-L model were higher than the ultimate tensile strength of resin cement. Tensile stress values and their concentration in the marginal part of the cement layer might indicate that clinical failure of the resin nanoceramic endocrown would occur in the cement, resulting in debonding, marginal leakage or secondary caries [48]. On the other hand, the smallest von Mises stress and tensile stress in the cement layer were seen in the E-Z and E-E models, respectively. This could be attributed to the high elastic modulus of these materials. The higher the elastic modulus of the material is, the more protection is provided for the adjacent structures, including the cement layer [31]. 

Although the findings of this study are essential to evaluate the biomechanical behavior of endocrowns from CAD/CAM materials, the results of this FE analysis should be considered carefully, since all structures were assumed to be isotropic and linearly elastic, while their real properties might be different. Moreover, the properties of the bonding to the restorative material and dentin may influence the type of failure. Despite the complexity of the oral environment, only static loadings were applied in this study to simplify the conditions required to perform the analysis. Although mandibular molars were selected as they lose hard tissues repeatedly, the type of the tooth may influence the pattern of stress distribution, since teeth are subjected to different forces in various magnitudes and directions. Furthermore, more studies about translucent zirconia and zirconia-reinforced lithium silicate are highly suggested to be performed to assess their mechanical properties, which are crucial for implementing some failure criteria. Further numerical studies are also needed to stimulate the nonlinearly elastic and nonhomogeneous properties of some components. The influences of other patterns of occlusal contacts, such as cross-bite, and different groups of teeth are also suggested to be studied.

## 5. Conclusions

Resin nanoceramic caused high stress concentration and displacement in dental structures, which might not make it a suitable material for endocrowns. It also caused high tensile stress in the cement layer with a high Mohr–Coulomb ratio in it, and that may compromise the unity of the endocrown–tooth complex. Translucent zirconia might be the best material for endocrowns to preserve the tooth–restoration complex since it absorbed stresses and showed low displacement within it and in the dental tissues. Lithium disilicate ceramic and zirconia-reinforced lithium silicate could be used to manufacture endocrowns as they offer an acceptable range of stresses, Mohr–Coulomb ratio, and displacement in the endocrown and dental structures. According to stress distribution and levels of stresses and displacements, dental materials with high elastic modulus appear to protect dental structures and the endocrown–tooth complex under occlusal loadings more than materials whose elastic moduli are low.

## Figures and Tables

**Table 1:** The models and their materials.

Model	Material	Abbreviation	Chemical Composition (%wt) *	Example	Manufacturer
E-E	Lithium disilicate glass ceramic	LDS	SiO_2_ (80.0), Li_2_O (19), K_2_O (13), P_2_O_5_ (11), ZrO_2_ (8), ZnO (8), Al_2_O_3_ (5), MgO (5) and coloring oxides	IPS e.max CAD	Ivoclar Vivadent GmbH, Germany
E-P	Polymer-infiltrated ceramic network	PICN	Ceramic part (86 wt%): SiO_2_ (63), Al_2_O_3_ (23), Na_2_O (6), B_2_O_3_ (2), ZrO_2_ (<1), CaO (<1). Polymer part (14 wt%): UDMA and TEGDMA	VITA Enamic	VITA Zahnfabrik, Germany
E-S	Zirconia-reinforced lithium silicate glass ceramic	ZLS	SiO_2_ (64), Li_2_O (21), K_2_O (4), P_2_O_5_ (8), Al_2_O_3_ (4), ZrO_2_ (12), CeO_2_ (4), La_2_O_3_ (0.1) and pigments (6)	VITA Suprinity	VITA Zahnfabrik, Germany
E-L	Resin nanoceramic	RNC	Nanomer and nanocluster fillers (nanoceramic material 80% wt). Nanoclusters (0.6–10 µm) of 20 nm silica and zirconia 4–11 nm.	Lava Ultimate	3M ESPE, USA
E-Z	Translucent zirconia	-	ZrO_2_, HfO_2_, Y_2_O_3_ (> 90), Al_2_O_3_ (<0.5) and other oxides (≤1) **	DD Bio zx2	Dental Direkt GmbH, Germany

* Chemical compositions are according to the manufacturers. ** (Schatz et al. [13]).

**Table 2:** The number of nodes and elements in the models.

Model *	Elements	Nodes
E-E	56,274	110,644
E-L	64,796	126,213
E-S	64,796	126,213
E-P	64,796	126,213
E-Z	50,626	100,495

* E-E: lithium disilicate glass ceramic; E-L: resin nanoceramic; E-S: zirconia-reinforced lithium silicate glass ceramic; E-P: polymer-infiltrated ceramic network; E-Z: translucent zirconia.

**Table 3:** The mechanical properties of the materials.

Material	Young’s Modulus (GPa)	Poisson Ratio	UTS * (MPa)	UCS ** (MPa)
Enamel	84	0.33	-	-
Dentin	18.6	0.30	-	-
Periodontal ligament	0.069	0.45	-	-
Resin cement	8.3	0.35	45	178
Cortical bone	13.7	0.30	-	-
Trabecular bone	1.37	0.30	-	-
Gutta percha	0.69	0.45	-	-
Translucent zirconia	210	0.307	745	904
Zirconia-reinforced lithium silicate ceramic	102.9	0.208	459 ^‡^	676 ^‡^
Lithium disilicate glass ceramic	83	0.21	173	448
Polymer-infiltrated network ceramic	30	0.23	100	370
Resin nanoceramic	12.7	0.47	100	516

* UTS: ultimate tensile strength. ** UCS: ultimate compressive stress. ^‡^ UTS and UCS are estimated.

**Table 4:** The Mohr–Coulomb ratio in endocrowns from all materials when axial and oblique loadings were applied *.

Model	Axial Loading	Oblique Loading
E-E	0.30	0.39
E-S **	0.14	0.15
E-L	0.48	0.70
E-P	0.52	0.68
E-Z	0.08	0.09

* Mohr–Coulomb ratio was calculated based on the peak of maximum and minimum principal stresses in the restorations. ** The ultimate tensile and ultimate compressive strengths of zirconia lithium silicate ceramic were estimated to be in a range between the strength of LDS and zirconia ceramic.

**Table 5:** The Mohr–Coulomb ratio in cement layer in the models when axial and oblique loadings were applied.

Model	Axial Loading	Oblique Loading
E-E	0.40	0.57
E-S	0.59	0.69
E-L	1.35	1.06
E-P	0.78	0.73
E-Z	0.57	0.75

## Data Availability

Data are available on request from corresponding author.
